# Muco-bioadhesive containing g*inger officinale* extract in the management of recurrent aphthous stomatitis: A randomized clinical study

**Published:** 2015

**Authors:** Parya Haghpanah, Ali Akbar Moghadamnia, Amin Zarghami, Mina Motallebnejad

**Affiliations:** 1 Department of Periodontology, School of Dentistry, Babol University of Medical Sciences, Babol, Iran.; 2 Department of Physiology & Pharmacology, Babol University of Medical Sciences, Babol, Iran.; 3 Student Research Committee, Babol University of Medical Sciences, Babol, Iran.; 4 Dental Materials Research Center, School of Dentistry, Babol University of Medical Sciences, Babol, Iran.

**Keywords:** Recurrent Aphthous Stomatitis, Ginger Officinale, Muco-bioadhesive, Herbal remedies

## Abstract

**Background:**

Recurrent aphthous stomatitis (RAS) is the most common oral mucosal lesions in the general population. Various treatment modalities have been used; but no specific therapy proved to be definitive*.* *Ginger Officinale* (ginger) indicated to have anti-inﬂammatory properties in herbal medicine. Thus, this study aimed to evaluate the efficacy of ginger containing bioadhesive in the treatment of aphthous ulcers.

**Methods:**

In this randomized double-blind placebo-controlled trial, 15 patients were enrolled. The clinical efficacy of the mucoadhessive on pain, inflammatory zone and ulcer's diameter in the test period was compared with that of the base treatment and no treatment periods during 10 days of study.

**Results:**

Significant reduction in pain was observed on day 5 between placebo (using base bioadhesives) and without treatment periods at the first phase of the study (4.53 vs. 3.27; P=0.038. ( Reduction in inflamed halo diameters was significant on day 1 between without treatment and ginger containing bioadhesives )46.73 vs 28.67; P=0.044). Other variables such as the diameter of ulcers did not indicate any significant differences in both periods.

**Conclusion:**

This study indicated that ginger bioadhesive is capable to relieve pain of RAS. However, its efficacy on ulcer diameter, inflamed halo and healing time was not significantly different compared to the results of the placebo received period.

Recurrent aphthous stomatitis (RAS) is one of the most common oral mucosal painful ulcerative lesions which can significantly affect the life quality of patients. This condition is associated with some pathological complications along with similar clinical demonstrations as round or elliptic recurrent lesions in oral mucosa (1-3). The prevalence of the disease depends on the race and socio-economic condition, but an overall 10-20% of the general population is affected by the disease. The onset of the RAS is usually in childhood and the severity and number of lesions will decreased with aging (4-6). Different studies have introduced different factors affecting the disease such as genetic predisposition, immune disorders, drugs, foods, hormonal changes, lack of vitamins and so on. However, the etiology of this illness is unknown (7-8). So far, many treatments have been introduced such as administration of antibiotics, anti-inflammatories, immune system modulators, traditional and home complementary remedies. 

Because of the broad spectrum of side effects following systemic administration of these agents, the topical dosage forms such as ointment, biopatch, mouthwash, and other local drug delivery systems are preferred (9, 10). In this regard, some studies have been conducted on the use of mucoadhesive as a drug delivery system to treat oral lesions, and according to the protective effect of this drug, it has been applied as an independent therapeutic method to relieve aphthous stomatitis (11, 12). Nevertheless, a few clinical evidence has been reported on the efficacy of different therapeutic methods. A part of this issue may result from the difficulty with real measuring the effectiveness of treatments for mucositis (6). 

Ginger is a native eastern Indian medicinal plant and it is one of the most common herbs in traditional medicine (13). Ginger has been used as an anti-inflammatory agent to treat arthritic disorders since many years ago. Some studies have also confirmed and demonstrated the anti-inflammatory effect of this plant (14, 15). Penna et al. have concluded that ginger can probably reduce skin edema associated with serotonin and carrageenan by its antagonistic effect on serotonin receptors significantly (16). On the other hand, this drug has been used as sedative and pain regulator which results from substance P release (17). However, few high quality studies have investigated the effectiveness of topically administered materials in various clinical conditions (18).

In the present study, a type of mucoadhesive has been introduced that contains natural elements available in Iranian traditional medicine. Hence, the study aimed to evaluate the effect of mucoadhesive containing ginger extract to relieve pain and other manifestations of recurrent aphthous stomatitis. 

## Methods

The present study was a double-blinded clinical trial. The protocol approved by Research Review Board, Babol University of Medical Sciences, Babol, Iran. The IRCT code of the study is: IRCT201311101760N27.

**Patient selection:** Fifteen patients were randomly selected. All patients had experienced minor aphthous within the past 2 months. They were evaluated during three aphthous periods for a minor aphthous stomatitis within the past 24h, confined to the lips and oral mucosa within each period. The subjects with major aphthous stomatitis and numerous minor stomatitis, or patients with lesions in the other areas of the lips and oral mucosa were excluded. Other exclusion criteria were: wearing a denture, receiving antibiotics for RAS and inﬂammatory and allergic conditions, smoking, pregnancy, estimation of poor cooperation during the study and unable to apply patches. Informed consent was taken for each participant. 

**Bioadhesive preparation:** Two types of mucoadhesive were provided. One was a mucoadhesive base without drug which was used as placeo (contains tragacanth gum, alcohol, sodium benzoate, and distilled water), and the other one was a mucoadhesive containing ginger’s alcoholic extract. In dry site, they are non-adherent and are easily applied to zone. Contact with saliva at the site, hydrated material, forming a sticky hydrogel which attaches to the mucosa at the lesion's site (19). All mucoadhesives were prepared in the Department of Pharmacology of Babol University of Medical Sciences. The prepared mucoadhesives were cut into circles with a diameter of 1cm, and were packed quaternary in a plastic small bag. The packages were encoded by the researcher in a way that both examiners and patients were blinded of the type of mucoadhesive in the envelope. 

**Clinical intervention: **The patients were asked to refer to the clinic during the first 24h after the onset of aphthous stomatitis. The ﬁrst episode of RAS was considered to record the baseline characteristics of aphthous in each subject. The patients completed this step and the baseline data for each subject was determined. For the first step of the study, the subjects were divided into two, placebo and ginger mucoadhesive receiving periods, respectively. The study was carried on in the next two periods of aphthous stomatitis episodes. The application of mucoadhesive was instructed to the subjects and they were asked to use it daily for 20 min after every meal and before going to bed. The treatment duration was 7 days and the subjects were examined during and after the treatment of aphthous stomatitis. The next step of the study was delayed up to the next RAS episode happened and the previous treatment method was washed out clinically. For the second step, the study population was carried on ginger. 

**Therapeutic evaluation:** The patients were asked to record the daily level of pain severity of the ulcers before meals and before bedtime through VAS (Visual Analogue Scale). The average diameter of the ulcer on days 0, 1, 3, 5, 7 of the study and accordingly the average diameter of the inflammatory zone in the mentioned days were recorded by the examiner. The diameter of lesions and inflammatory zone were measured and individually recorded for each patient. In the last observation session, the duration of decreasing inflammatory zones and elimination of lesions as final treatment of aphthous stomatitis were approved and recorded by the examiner. 

**Statistical analysis:** Repeated Measures, Friedman, Mann-Whitney, one-way ANOVA and post hoc (Scheffe) tests were used. The difference between data was considered statistically significant at p-value lower than 0.05.

## Results

The study group consisted of 10 men and 5 women with an average age of 22.86±2.25 years (17-37 years). No side effect which could restrict the progression of treatment was reported by the cases who applied mucoadhesive ginger and all participants finished three periods of the study.

**Figure 1:**
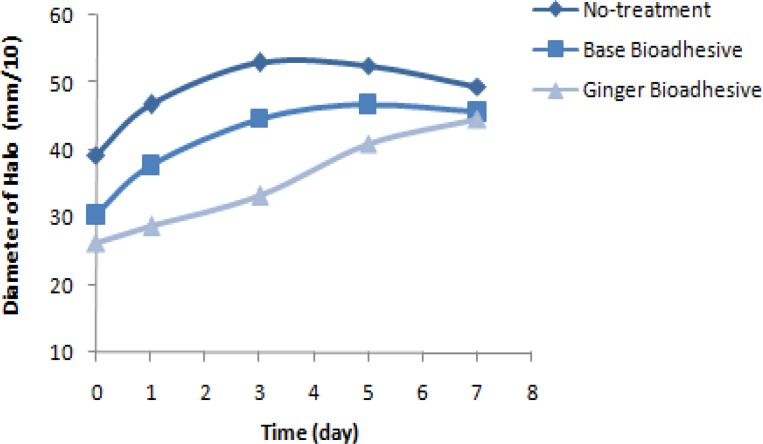
Trend of pain intensity based on VAS during day 0–10 of study in three different periods of the treatment

According to the results of the repeated measures test, the average of pain severity based on VAS method with different times of measurement in mucoadhesive received period was significantly different every day of the three studied periods (p<0.001). However, the severity of pain showed a significant decrease only on the fifth day of placebo-treated with mucoadhesive, compared to that of without treatment period (P=0.038) (figure 1). 

**Figure 2:**
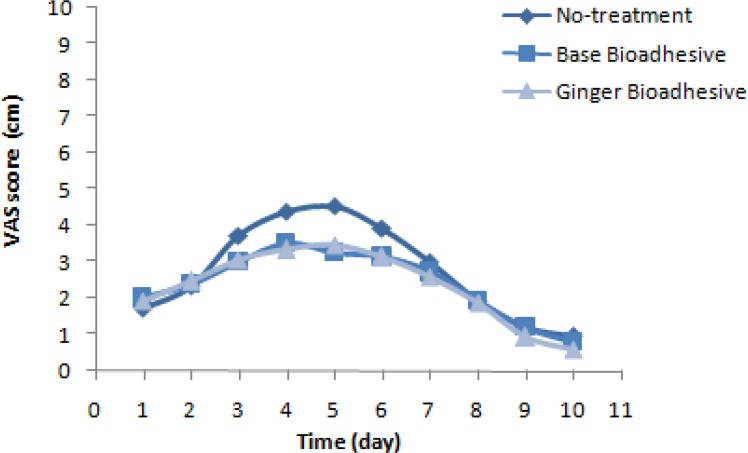
Trend of inflammatory zone on days 1, 3, 5 and 7 of observation in three different periods of treatment

**Figure 3:**
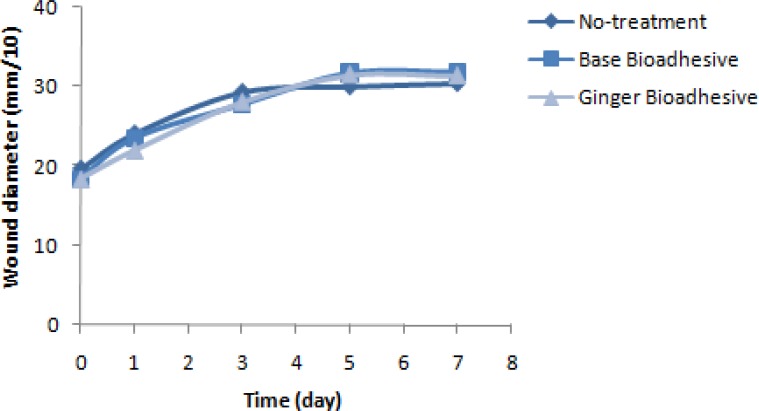
Trend of wound diameter on days 1, 3, 5 and 7 of observation in three different periods of treatment

According to figures 2 and 3 that illustrate the results of Friedman test, the average diameter of inflammatory and lesion zones showed significant difference in intra-group comparisons daily of the three periods of the study (P=0.045); also, in ginger received episode, a significant decrease in the diameter of inflammatory zone was observed on the first day of treatment with ginger in comparison with the period without treatment (p<0.05). The mean and SD of pain relief duration (day) in the three periods of ginger, placebo and without treatment received was 14.2±3.21, 15.13±3.44, and 13.87±4.22, respectively. Therefore, no significant difference was observed in pain relief duration among the three periods of treatment (p=0.351).

## Discussion

The present study evaluated the effect of mucoadhesives containing ginger extract on the treatment of recurrent aphthous stomatitis, and the diameter of inflammatory zone and lesion, pain severity and healing process on the first, third, fifth and seventh days of study between placebo and ginger mucoadhesive receiving periods were compared. According to the results, a significant difference was observed among daily pain severity of cases within the three studied periods. However, there was a significant decrease among the pain severity of cases only on the fifth day of treatment with placebo rather than that of the period of without treatment. This result is in consistent to the results of the other studies conducted on the maximum effectiveness of cellulose based hydrogel mucoadhesive on the first days of the treatment (20, 21). It seems that the anti-inflammatory effect of ginger can improve the clinical symptoms of aphthous stomatitis.

Topical drug delivery systems have been significantly developed and are widely applied in the treatment of mucosal lesions. Local drug delivery systems, such as mucoadhesive, have been widely developed regarding the treatment of oral lesions and local anesthetics, in addition to the gels and ointments. Since in the current mentioned system, direct application of drug on oral lesions has been extended, it seems that this system is more efficient to reach the therapeutic goal (22). 

Moghaddamnia et al. have also reported that the mucoadhesive significantly reduces the pain severity of aphthous stomatitis, with or without liquorice (19). Martin et al. showed that the size of lesion has significantly decreased in the experimental group on the eighth day of treatment with mucoadhesive containing liquorice extract (23). Shemer et al. have also reported that the cyanoacrtylate-2-octyle mucoadhesive can significantly relieve the lesions caused by suturing, and reduce the duration of treatment and its clinical symptoms in comparison with the application of regular monofilaments (24).

The results of some former studies revealed that the covering property of mucoadhesive may reduce the pain and shorten the treatment period of aphthous stomatitis (25-27). In these studies in which cyanoacrylate-2-octyle mucoadhesive was applied, a significant decrease in the severity of pain, healing duration and also the size of lesion was observed. But the main problem for the use of cyanoacrylates and hydroxyl propyl cellulose is that the production of their mucoadhesive is costly. Hence, new research has been conducted on the production of mucoadhesives from less expensive natural elements. One of the advantages of the current study was that natural elements used in the preparation of mucoadhesive which was economical, considering the financial constraints on the process of production. 

In a study conducted by Moghaddamnia et al. on the formulation and production of alum containing mucoadhesive, through several examinations, a different base of carboxymethyl cellulose (CMC) and tragacanth (gum Arabic) was prepared, and the examinations regarding the release of alum as an effective substance with the use of proper texture and water absorption through gingival were performed. 

The results of the study indicated that the CMC based mucoadhesive was more suitable considering the more effectiveness and ease of application, and were preferred by the first users; although gum-based adhesives had more mucosal adhesion (28). 

Motallebnejad et al. also evaluated the effect of a type of tragacanth based mucoadhesive on aphthous stomatitis, and then studied its mucoadhesive effect associated with triamcinolone acetonide on aphthous stomatitis; they finally concluded that using mucoadhesive reduces the pain severity of aphthous stomatitis and reported that triamcinolone acetonide has no effect on the duration of treatment. In this regard, mucosal adhesion of mucoadhesive in all cases was more than 20 min (29). 

We had limitations in this study in regard to the small sample size and duration of the study period which require future RCT studies in larger populations. Since aphthous has recurrent characteristics, so the importance of long- term treatment program may exert additional benefits.

In Conclusion considering the results of the present study, ginger-containing mucoadhesive can reduce the severity of pain, but did not show any significant effect on the diameter, zone of lesion and duration of treatment. 

## Notes

**Funding:** The authors thank the Deputy of Research and Technology of Babol University of Medical Sciences for financially supporting the project. 

## Notes

**Trial Registry: **IRCT201311101760N27 Available at: www.IRCT.ir
